# A System of Medicine

**Published:** 1899-11

**Authors:** 


### BOOK REVIEWS, PAMPHLETS, EXCHANGES.

### BOOK REVIEWS.

[Contributions solicited for review.]

A System of Medicine. By many writers. Edited by T. Clifford Allbutt, M.A., M.D., LL.D., F.R.C.P., etc., vol. vi., pp. 944. Price $5. Macmillan Co.

The sixth volume of this excellent system has recently been issued. It concludes the consideration of the Diseases of the Circulatory System, discusses Diseases of Muscles, and fairly enters upon the subject of Nervous Diseases. To Dr. Newton Pitt has been allotted Right-sided Valvular Diseases; to Sir R. Douglas Powell Angina Pectoris; to Dr. F. T. Roberts the Mediastinum and Thymus Gland; to Mr. H. H. Clutton, Phlebitis; to Dr. F. W. Mott, Arterial Degeneration and Diseases; to Sir W. Gairdner, Aneurism of the Aorta, and to Dr. Rolleston, Aneurism of the Abdominal Arteries. As this is almost entirely a British System of Medicine Dr. Welch, of Johns Hopkins University, is especially to be congratulated upon having been chosen to write the articles upon Thrombosis and Embolism. Dr. Batten has written on Myositis; Dr. Hale White on Myotonia Congenita (said to be the rarest disease known); Dr. Beevor on Idiopathic Muscular Atrophy and Hypertrophy; Dr. Aldren Turner on Facial Hemiatrophy and Hemihypertrophy, and on Diseases of the Cranial Nerves; Drs. Sherrington and Sharkey on Tremor, Tendon-phenomenon and Spasm; Dr. Turney on Neuropathic Affections of Bones and Joints; Mr. Hopkins on Neuropathic Diseases of Soft Tissues; the editor on Adiposis Dolorosa; Dr. Barlow on Raynaud's Disease and Erythromelalgia; Drs. Gibson and Fleming on Diseases of the Spinal Nerves; Dr. Judson Bury on Multiple Symmetrical Peripheral Neuritis; Dr. Head on Trigeminal Neuralgia; Dr. Brudenell Carter on Medical Ophthalmology; Mr. Horsley on Diseases of the

Vertebral Column, Tumors, Compression Palsies, and Dr. Risien Russell on Affections of the Spinal Meninges.

Besides four plates, the volume contains forty-four illustrations, and two tables. Many of the above authors are as well-known on this as on the other side of the Atlantic, and in the small space at our disposal we need only say that we look upon this system of medicine as exceedingly valuable, and worth far more than its price to all who are really interested in keeping abreast of the times. Two other volumes are still to come. A. W. S.

---

A QUIZ COMPEND OF GYNECOLOGY. By W. H. Wells, M.D., Adjunct Professor of Obstetrics and Diseases of Infancy in the Philadelphia Polyclinic. P. Blakiston's Sons & Co., Philadelphia. Price 80c.

This is a second edition of the Compend on Gynecology, and like all of the series is thoroughly up to date. The Messrs. Blakiston have given to the medical student a most excellent series of Compends. This little book is well illustrated, and, for its size, is exceedingly thorough. As a Compend it is exceedingly meritorious. R.

